# Antidiabetic Agents for Treatment of Parkinson’s Disease: A Meta-Analysis

**DOI:** 10.3390/ijerph17134805

**Published:** 2020-07-03

**Authors:** Shu-Yi Wang, Shey-Lin Wu, Ta-Cheng Chen, Chieh-Sen Chuang

**Affiliations:** 1 Division of Endocrinology and Metabolism, Department of Internal Medicine, Changhua Christian Hospital, Changhua 500209, Taiwan; 86761@cch.org.tw; 2 Department of Neurology, Changhua Christian Hospital, Changhua 500209, Taiwan; 14132@cch.org.tw (S.-L.W.); 67482@cch.org.tw (T.-C.C.); 3 College of Medicine, Kaohsiung Medical University, Kaohsiung 807378, Taiwan

**Keywords:** antidiabetic agent, Parkinson’s disease, meta-analysis, glucagon-like peptide 1, Thiazolidinedione

## Abstract

*Background*: Clinical and epidemiological studies suggest that two of the most common geriatric diseases, type 2 diabetes and Parkinson’s disease (PD), are linked. These studies notably suggest that treatment of insulin resistance in type 2 diabetes may beneficially modify the pathophysiology of PD and help to maintain motor and nonmotor function. In this meta-analysis, we evaluate the efficacy of new antidiabetic agents in the treatment of PD. *Methods*: We systematically searched PubMed, Medline, ProQuest, ScienceDirect, ClinicalKey, and Cochrane Library from the date of their inception until 15 March 2020. Multiple efficacy parameters were compared between treatment groups. The results are expressed as mean differences with 95% confidence intervals (CIs) in a random-effects model. *Results*: A meta-analysis of the data extracted from three randomized control trials revealed that treatment with exenatide yielded significant improvements in scores on the Unified Parkinson’s Disease Rating Scale Part I (UPDRS-I) (−0.438, 95% CI, −0.828 to −0.048, *p* = 0.028), UPDRS Part IV (UPDRS-IV) (−0.421, 95% CI, −0.811 to −0.032, *p* = 0.034) and the Mattis Dementia Rating Scale (MDRS) (−0.595, 95% CI, −1.038 to −0.151, *p* = 0.009). At the 12-month follow-up, the UPDRS Part III (UPDRS-III) scores in the off-medication phase revealed significant improvements in patients using exenatide (−0.729; 95% CI, −1.233 to −0.225, *p* = 0.005). Treatment with pioglitazone did not yield significant improvements in UPDRS, MDRS, or Parkinson’s Disease Questionnaire scores. *Conclusion*: This meta-analysis suggests that exenatide use is associated with the alleviation of cognitive, motor and nonmotor symptoms. However, long-term studies with a large sample size of patients with PD of varying severity are required.

## 1. Introduction

Parkinson’s disease (PD) is the most common movement disorder and the second most common neurodegenerative disease after Alzheimer’s disease (AD) among older adults [1]. PD prevalence is higher among older age groups and affects 1% of the population aged 60 years or older [2]. The cardinal motor features of PD include bradykinesia, rigidity, tremors, and postural instability resulting in gait disturbances. Several nonmotor symptoms complicate the course of illness, including sleep disorders, cognitive impairment, depression, autonomic dysfunction, and hyposmia. Therapeutic approaches are aimed at dopamine replacement and focus on restoring dopaminergic activity to control motor symptoms. Although these treatments can initially relieve symptoms, complex motor fluctuations and dyskinesias can develop over time, affecting the patient’s quality of life and mobility.

Previous studies have shown that people with type 2 diabetes have an increased risk of PD [3]. A high rate of abnormal glucose tolerance is observed among patients with PD, and studies have shown insulin resistance to be a pathologic driver of PD [4,5]. Therefore, antidiabetic agents may aid the management of PD through disease modification by targeting the underlying pathophysiological mechanisms. Incretins are small hormonal peptides that can stimulate pancreatic beta cells to regulate insulin release after eating. The incretin hormone glucagon-like peptide 1 (GLP-1) is best known for its effects on glucose homeostasis and regulation of insulin signaling [6]. GLP-1 analogues, activating the GLP-1 receptor (GLP1R), have been developed for the treatment of type 2 diabetes mellitus. GLP1R is expressed not only in the pancreas, but also in several other organs, such as the lungs, stomach, intestines, kidneys, heart, and brain [7,8]. Accumulating evidence suggests that these GLP-1 analogues can cross the blood-brain barrier to influence several neuronal pathways, such as those responsible for neuroinflammation and mitochondrial function [9]. Several studies have also demonstrated the neuroprotective effects of GLP1R stimulation in PD models, resulting in improvements in motor and nonmotor disorders [6]. Thiazolidinediones (TZDs) are a class of oral antidiabetic drugs that improve glycemic control in patients with type 2 diabetes by increasing insulin sensitivity [10]. They activate peroxisome proliferator-activated receptors and reduce insulin resistance in adipose tissue, muscles, and the liver [11]. Preclinical and early clinical studies suggest that TZDs exert neuroprotective effects in PD and other neurodegenerative diseases [12].

The purpose of this meta-analysis was to evaluate the effects of antidiabetic medications on motor and nonmotor symptoms in patients with PD. 

## 2. Materials and Methods

**Figure 1:**
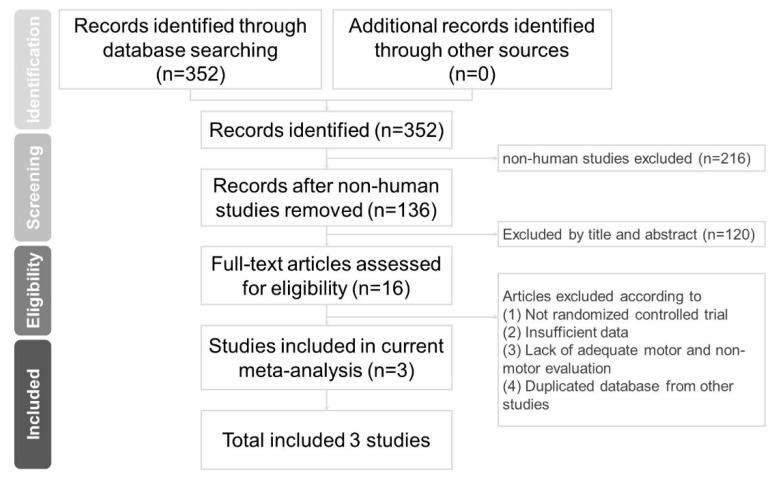
Flowchart of study selection.

This systematic review and meta-analysis followed the Preferred Reporting Items for Systematic Reviews and Meta-Analyses (PRISMA) reporting guidelines (Figure 1) [13].

### 2.1. Literature Search and Screening

PubMed, Medline, ProQuest, ScienceDirect, ClinicalKey, and Cochrane Library were systematically searched from the date of their inception until March 15, 2020, using the following searches: (“thiazolidinedione” or “glitazone”) and “Parkinson’s disease”, (“glucagon-like peptide 1” or “GLP-1”) and “Parkinson’s disease”, (“sodium-glucose cotransporter 2” or “SGLT-2”) and “Parkinson’s disease”, (“dipeptidyl peptidase-4 or TPP-4”) and “Parkinson’s disease”, (“gastric inhibitory polypeptide” or “GIP”) and “Parkinson’s disease”, “exenatide” and “Parkinson’s disease”, “pioglitazone” and “Parkinson’s disease”, and “liraglutide” and “Parkinson’s disease”. This search strategy was supplemented with manual searches of reference lists for eligible articles and recent reviews. After removing duplicate studies, we screened the titles and abstracts to evaluate article eligibility, upon which a list of potentially relevant studies for a full-text review was based. Only human randomized controlled trial (RCTs) articles were included. We eliminated nonclinical trials such as case series and observational studies. Two investigators (SY Wang and SL Wu) independently screened the titles and abstracts of the retrieved references for eligibility, and extracted relevant data from the articles. Where discrepancies arose, a third author (CS Chuang) was involved. Two authors (TC Chen and SL Wu) independently assessed the risk of bias among the included studies. Studies were then further classified in the overall risk of bias category.

### 2.2. Outcome Assessment

The results on the Unified Parkinson’s Disease Rating Scale (UPDRS) Parts I, II, III, and IV (UPDRS-I, UPDRS-II, UPDRS-III, UPDRS-IV), Parkinson’s Disease Questionnaire (PDQ-39), and Mattis Dementia Rating Scale (MDRS) were compared; other data unsuitable for meta-analysis were also reviewed in our study. The UPDRS is a widely used clinical tool that assesses functional status and motor performance [14]. The UPDRS-I measures nonmotor experiences involving mental status, behavior, and mood. The UPDRS-II measures activities of daily life. The UPDRS-III measures motor function, specifically assessing speech, facial expression, rigidity, finger taps, hand movement, leg agility, the ability to rise from a seated position, gait, posture, postural stability, bradykinesia, and action or postural tremor. Finally, the UPDRS-IV measures complications of therapy [15,16]. The UPDRS is commonly used as an international standard, and each criterion is scored from 1 to 5. Higher UPDRS scores indicate more severe PD. This scale has been demonstrated to be reliable and valid [15]. The PDQ-39 is a 39-item, self-reporting questionnaire that assesses how often patients with PD experience difficulties in eight aspects of daily life. PDQ-39 ranges from 0 to 100, with a higher score indicating a lower quality of life [17]. The MDRS, a widely used dementia screening instrument, generates subscale scores in five areas: attention, initiation-perseveration, construction, conceptualization, and memory. In this case, higher scores indicate a more severe condition [18]. It is widely used in screening for dementia in patients with PD. Changes in UPDRS, PDQ-39, and MDRS scores from baseline measurements were used to measure improvement in PD symptoms. 

### 2.3. Statistical Analysis

Because heterogeneity among the included studies was presumed, the data were analyzed using random-effects meta-analysis models—rather than fixed-effects models—in Comprehensive Meta-Analysis Software Version 3 (Biostat, Englewood, NJ, USA). Because all the input data were based on the same rating scales, we calculated the mean differences (MDs) in outcomes to provide clear and relevant information for clinicians. MDs of changes in PD symptoms between groups were analyzed using the Hedge *g* and 95% confidence intervals (CIs). We identified the heterogeneity between studies using Cochrane’s Q statistics (chi-square), inverse variance (*I^2^*), and *p* values. An *I^2^* value higher than 75% indicated high heterogeneity between the studies. We examined publication bias with funnel plots and the Egger regression test [19]. Statistical significance was set at a two-tailed alpha level of 0.05.

## 3. Results

### 3.1. Search Results

Three RCTs with 308 participants and an average age of 57.8 years were included [20,21,22]; 85.7% of the participants were men (Table 1). The average duration of symptoms at baseline was four years (2–11 years). The baseline UPDRS-III scores in the off-medication phase ranged from 15 to 34. 

### 3.2. Changes in UPDRS-I Scores

**Figure 2:**
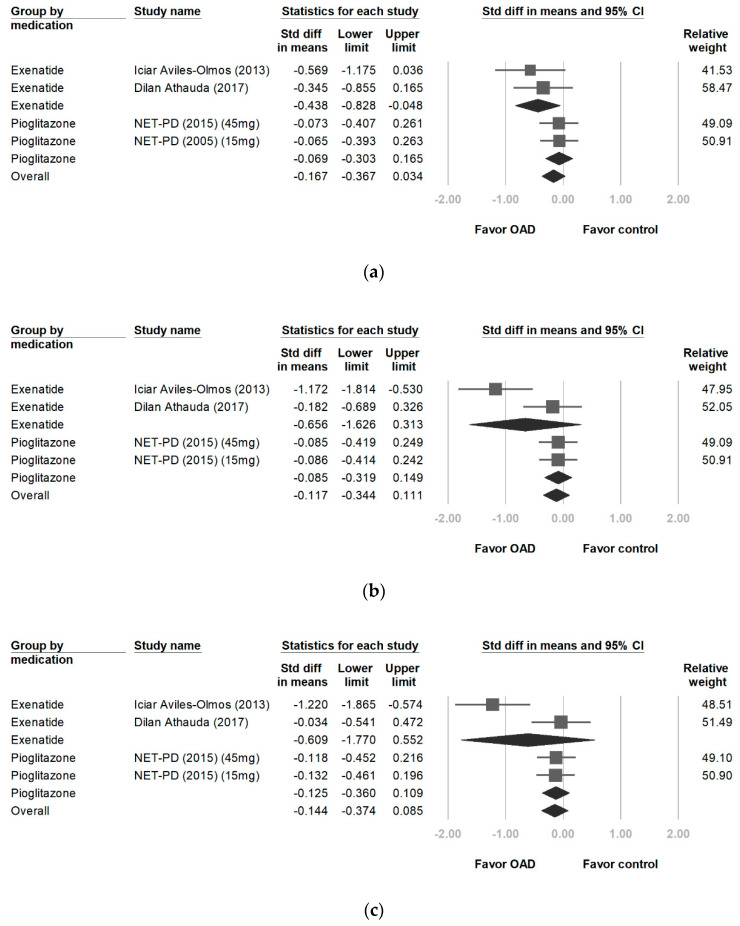

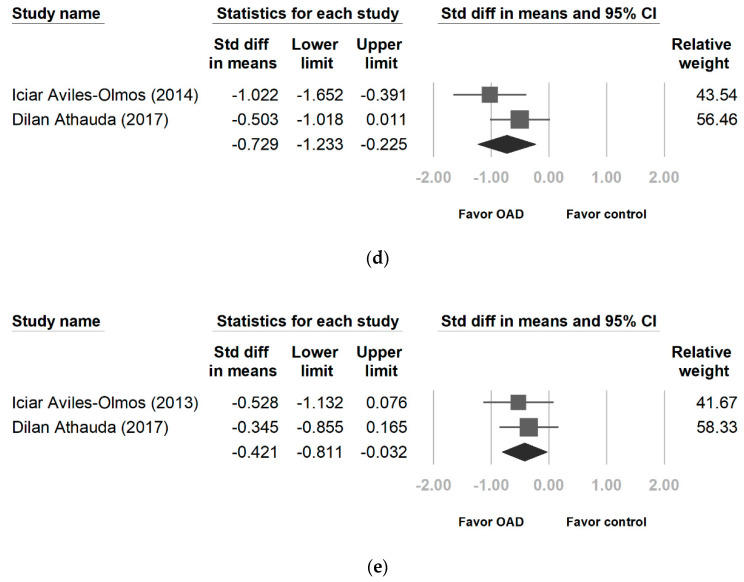
Forest plot of effect sizes for UPDRS-I (**a**) (significant improvement on exenatide: −0.438, *p* = 0.028), UPDRS-II (**b**), UPDRS-III during the on-medication phase (**c**), UPDRS-III at the 12-month follow-up during the off-medication phase (**d**) (significant improvement on exenatide: −0.729, *p* = 0.005), and UPDRS-IV (**e**) (significant improvement on exenatide: −0.421, *p* = 0.034). UPDRS: Unified Parkinson’s Disease Rating Scale.

A significantly greater improvement in UPDRS-I scores was observed among patients on exenatide than those in the control groups (−0.438, 95% CI, −0.828 to −0.048, *p* = 0.028). *I^2^* analyses revealed no significant differences in heterogeneity between treatment groups (*p* = 0.578, *I^2^* = 0%). However, pioglitazone did not yield a significant improvement (−0.069, 95% CI, −0.303 to 0.165, *p* = 0.564; Figure 2a).

### 3.3. Changes in UPDRS-II Scores

Exenatide users showed no significant improvements in UPDRS-II scores (−0.656, 95% CI, −1.626 to 0.313, *p* = 0.184). Significant differences in heterogeneity were observed between treatment groups (*p* = 0.018 and *I^2^* = 82.2%). No significant differences in UPDRS-II scores were observed between pioglitazone and placebo users (−0.085; 95% CI, −0.319 to 0.149; *p* = 0.475; Figure 2b).

### 3.4. Changes in UPDRS-III Scores

The use of exenatide did not yield significant improvements in UPDRS-III scores during the on-medication phase, with a score difference of −0.609 from baseline (95% CI, −1.770 to 0.552, *p* = 0.304). *I*^2^ analyses revealed significant differences in heterogeneity between treatment groups (*I*^2^ = 87%, *p* = 0.005; Figure 2c). No significant differences in UPDRS-III scores were observed between the pioglitazone and control groups (−0.125, 95% CI, −0.360 to 0.109, *p* = 0.218). The patients with PD were followed for 12 months after cessation of the trials [22,23]. The off-medication UPDRS-III scores improved by −0.729 (95% CI, −1.233 to −0.225, *p* = 0.005; Figure 2d). *I^2^* analyses revealed no significant differences in heterogeneity between treatment groups (*I^2^* = 35.8%, *p* = 0.212).

### 3.5. Changes in UPDRS-IV Scores

Exenatide users showed significantly greater improvements in UPDRS-IV scores than those in the control group (−0.421, 95% CI, −0.811 to −0.032, *p* = 0.034). *I*^2^ analyses revealed no significant differences in heterogeneity between treatment groups (*I*^2^ = 0%, *p* = 0.650; Figure 2e).

### 3.6. Changes in MDRS Scores

**Figure 3:**
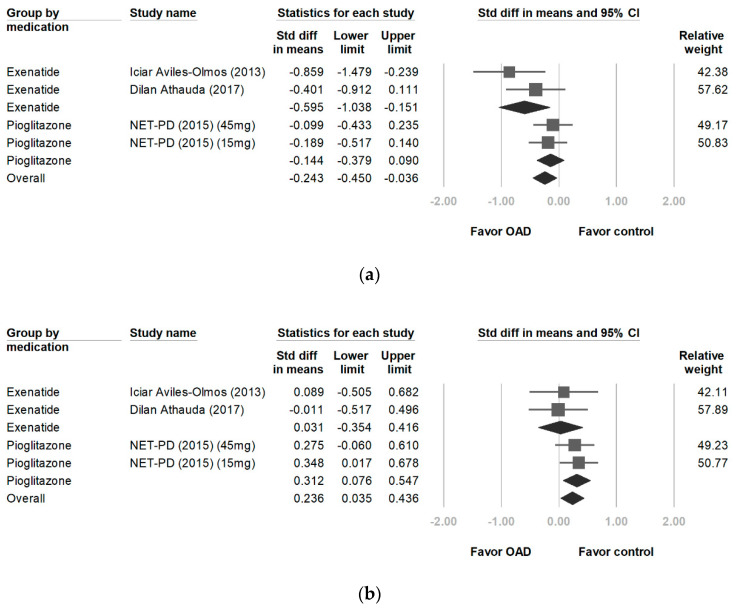
Forest plot of effect sizes for MDRS (**a**) (significant improvement on exenatide: −0.595, *p* = 0.009), and PDQ-39 (**b**). MDRS: Mattis Dementia Rating Scale, PDQ-39: Parkinson’s Disease Questionnaire.

The patients receiving exenatide had significantly larger improvements in MDRS scores than those in the control group (−0.595, 95% CI, −1.038 to −0.151, *p* = 0.009). Heterogeneity was not statistically significant (*p* = 0.264 and *I^2^* = 19.8%). No significant differences were observed between the pioglitazone and control treatments (−0.144, 95% CI, −0.379 to 0.090, *p* = 0.227; Figure 3a).

### 3.7. Changes in PDQ-39 Scores

No significant differences in PDQ-39 scores were observed between the exenatide and placebo groups (0.031, 95% CI, −0.354 to 0.416, *p* = 0.874). No significant differences in heterogeneity were observed between treatment groups (*p* = 0.803, *I^2^* = 0%). The patients receiving pioglitazone had significantly higher PDQ-39 scores than those in control group (0.312, 95% CI, 0.076 to 0.547, *p* = 0.009; Figure 3b).

### 3.8. Quality Assessment

**Figure 4:**
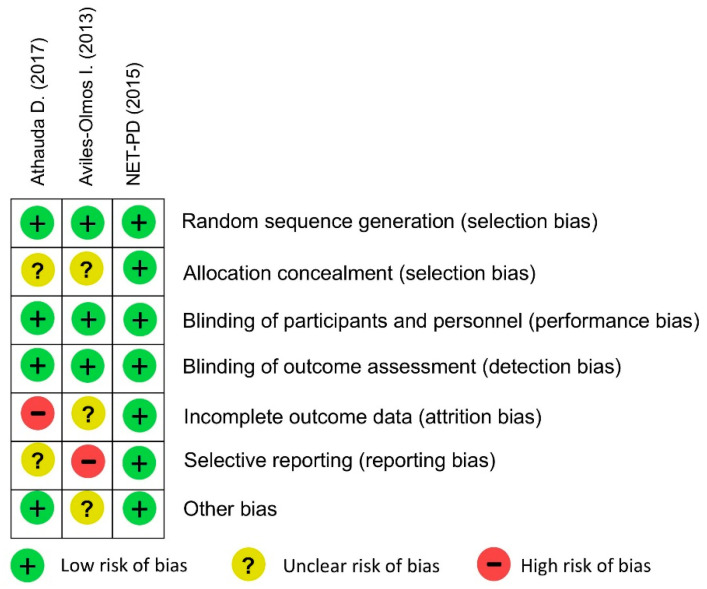
Quality assessment for the risk of bias for each study included in this meta-analysis.

Figure 4 shows the risk of bias for each study included in this meta-analysis. Quality assessment in this meta-analysis demonstrated a low risk of bias in NET-PD study. The biases in the two studies involved incomplete outcome data (attrition bias) and selective reporting (reporting bias).

## 4. Discussion

This meta-analysis discovered that the use of exenatide was associated with improvements in cognitive test scores, quality of life, and reductions in nonmotor symptoms and motor complications in patients with PD; however, pioglitazone treatment yielded no such improvements. In two studies, the patients were followed up for 12 months after completion of the trial. The UPDRS-III scores in the off-medication phases reflected significant improvements in the motor function of patients using exenatide. 

Chronic systemic inflammation and impaired mitochondrial metabolism play a role in the development of type 2 diabetes and the neurodegenerative disease. The coexistence of dopaminergic neurons and insulin receptors in the substantia nigra reinforce the occurrence of a direct association between DM and PD. Dopamine depletion in the striatum is related with the decrease of insulin signaling in basal ganglia. It was found that peroxisome proliferator-activated receptor-γ, GLP-1 and dipeptidyl peptidase-4 are important therapeutic targets for PD.^3,4,5^ Several in vivo and in vitro studies have reported neuroprotective effects of GLP1R agonists in experimental models of PD and AD [24]. In the PD models, GLP1R agonists reduced the expression of proinflammatory cytokines and the degeneration of dopaminergic neurons, prevented cognitive impairment, and prolonged lifespan [25,26,27,28]. This might partially explain why GLP1R agonists led to improvements in MDRS scores for cognitive function in patients with PD. 

UPDRS is the most widely used PD rating scale. UPDRS-IV is used to assess two motor complications, namely, dyskinesia and motor fluctuation. Dyskinesia in patients with PD, often referred to as levodopa (L-dopa)-induced dyskinesia, can be described as uncontrolled jerking, dance-like movements, wiggling, or twitching. The pattern of dyskinesia varies with the time of onset in relation to L-dopa intake. 

Peak-dose dyskinesia occurs when plasma levels of L-dopa are high, and tends to be predominantly chorea with occasional dystonia [29]. The short half-life of L-dopa, i.e., approximately one and a half to two hours, leads to alternating peaks and troughs of plasma levels, and is believed to play an important role in the development of dyskinesia [30]. The depletion of dopaminergic neurons also appears to be crucial to the pathophysiology of L-dopa-induced dyskinesia [31,32]. Dyskinesia is more prevalent in more advanced PD, which is accompanied by greater dopamine terminal loss [33]. The GLP-1 receptor is widely expressed in various brain regions such as the brainstem and hypothalamus [34]. GLP-1 can readily cross the blood-brain barrier and regulates glucose homeostasis as well as brain metabolism [35]. Several studies have demonstrated the neuroprotective and neurotrophic effects of GLP1R agonists [36,37]. Experimental models have shown that GLP1R agonists normalize dopamine release, induce mitochondrial biogenesis, reduce neuroinflammation, and improve control of synaptic plasticity [6,27,38]. This may partially explain the role it has in reducing dyskinesia and motor fluctuation in patients with PD. 

A secondary analysis of the exenatide clinical trial in PD data has reported changes of molecules released by neurons that can be detected in a blood sample [39]. Neurons can release small vesicles containing a range of different messenger proteins as a means of communicating with other cells. Exosomes are a type of extracellular vesicle; they can cross the blood-brain barrier and carry messenger proteins in the blood. This analysis provides biomarker evidence that peripherally administered exenatide may normalize brain insulin signaling in association with the activation of protein kinase B (Akt) and mechanistic target of rapamycin (mTOR) cascades in PD. The signaling molecules involved in insulin-related pathways are also related to the promotion of neuronal survival.

Our study has several limitations. First, the severity of PD in the patients was mild to moderate. The Hoehn and Yahr Scale was used to measure disease progression and level of disability. The participants in the included studies were in stages 0 to 2.5 according to the Hoehn and Yahr Scale. Future studies should include patients with moderate to severe PD to determine whether GLP1R agonists also alleviate PD symptoms in such patients. Second, the follow-up time was short; thus, the long-term effects of GLP1R agonists could not be inferred. Future studies should use a longer follow-up period to determine whether GLP-1 receptor agonists continue to ameliorate PD symptoms. Moreover, as in most meta-analyses, another limitation of the current study was the heterogeneity of the included studies in terms of their duration, initial severity of PD, drug dosage, different concomitant medications for PD, and the wide variety of ages of the patients.

## 5. Conclusions

The current meta-analysis provides evidence that exenatide use is associated with alleviation of cognitive, motor, and nonmotor symptoms. However, long-term RCTs with a large sample size and varying severity of PD patients are required to investigate the efficacy, safety, and tolerability of treatment with antidiabetic agents.

## Figures and Tables

**Table 1:** Summary of study characteristics in this meta-analysis.

Author (year)	Medication	Subjects	Mean Age	Males (%)	Duration of Symptoms at Baseline, year	Baseline UPDRS III off Medication	Hoehn-Yahr Stage 1–2/2.5 (n)
Aviles-Olmos I. (2013)	Exenatide (2mg) Placebo	20 24	61.4 ± 6.0 59.4 ± 8.4	15(75%) 20(83%)	9.6 ± 3.4	31.0 ± 11.2	14/6
					11.0 ± 5.9	34.0 ± 15.0	16/8
Athauda D. (2017)	Exenatide (2mg) Placebo	31 29	61.6 ± 8.2 57.8 ± 8.0	22(71%) 22(76%)	6.4 ± 3.3	32.8 ± 9.7	29/2
					6.4 ± 3.3	27.1 ± 10.3	29/0
NET-PD (2015)	Pioglitazone (15mg) Pioglitazone (45mg)	71 63	61.3 ± 10.6 58.8 ± 9.2	53(74%)	2.3 ± 1.9	17.1 ± 7.7	71/0
				47(70%)	2.0 ± 1.2	15.0 ± 7.1	63/0
	Placebo	70	50.8 ± 9.9	48(68%)	2.3 ± 2.3	15.3 ± 6.5	70/0

Data are mean (SD) or n (%).
